# Use of the Phylobone database for the annotation of bone extracellular matrix proteins in reindeer (*Rangifer tarandus*)

**DOI:** 10.1177/00368504241244666

**Published:** 2024-04-13

**Authors:** Alba Sánchez-Reverté, Margalida Fontcuberta-Rigo, Miho Nakamura, Pere Puigbò

**Affiliations:** 1 Medicity Research Laboratory, Faculty of Medicine, 8058 University of Turku, Turku, Finland; 2 Department of Biochemistry and Biotechnology, University Rovira i Virgili, Tarragona, Catalonia, Spain; 3 Institute of Biomaterials and Bioengineering, Tokyo Medical and Dental University, Chiyoda, Tokyo, Japan; 4 Graduate School of Engineering, Tohoku University, Sendai, Miyagi, Japan; 5 Department of Biology, 8058 University of Turku, Turku, Finland; 6 303231 Eurecat, Technology Center of Catalonia, Nutrition and Health Unit, Reus, Catalonia, Spain

**Keywords:** Extracellular matrix, proteins, bone, database, cervidae, *Rangifer tarandus*

## Abstract

Bone extracellular matrix (ECM) proteins play a key role in bone formation and regeneration, including structural and regulatory functions. The Phylobone database consists of 255 ECM protein groups from 39 species and can be used to support bone research. Here, we gathered bone ECM proteins from reindeer (*Rangifer tarandus*), a member of the Cervidae family. The importance of reindeer lies in their ability to regenerate their antlers, in both male and female individuals. Protein sequences were extracted from the National Center for Biotechnology Information's repository and selected by homology searches. We identified 215 proteins and their corresponding functional domains, which are putatively present in the bone ECM of reindeer. Protein sequence alignments have shown a high degree of conservation between *R. tarandus* and other members of the Cervidae family. This update expands the Phylobone database and shows that it is a useful resource for the preliminary annotation of bone ECM proteins in novel proteomes.

## Highlights

- Identification of 215 putative bone extracellular matrix (ECM) proteins in *Rangifer tarandus*.
- Current version of the Phylobone database contains seven species of the Cerevidae family.
- Phylobone is a reliable resource for pre-annotations of bone ECM proteins in novel organisms.

## Introduction

The bone extracellular matrix (ECM) environment is composed of organic and inorganic compounds, including collagen and hydroxyapatite. In addition to collagen, the organic portion includes a large variety of noncollagenous proteins.^1^ ECM proteins have both structural and regulatory properties that support flexibility and cell signalling to the bone tissue.^2^ They are also involved in bone formation and regeneration through the regulation of cell adhesion, proliferation, differentiation and bone mineralization.^1,3^ The involvement of bone ECM proteins in these processes makes them a potential target for the study and treatment of osteoporosis.

Recently, our research group has been working on the Phylobone project to study bone ECM proteins in human and model species.^4^ The first version of the Phylobone database contains a functional and phylogenetic characterization of 255 protein groups from 39 species of vertebrates and invertebrates. This database is a useful resource for studying the bone ECM proteins involved in bone formation and regeneration in the most common animal models, such as mouse (*Mus musculus*), rat (*Rattus norvegicus*), zebrafish (*Danio rerio*) and frog (*Xenopus laevis*)*.* The database provides information on protein functions, domains and protein–protein and protein–drug interactions and includes several links to external databases, including InterPro, UniProt, DrugBank and KEGG.^4^ Moreover, the phylobone database is a reliable resource for a pre-annotation of putative bone ECM proteins in novel proteomes.

**Figure 1.**
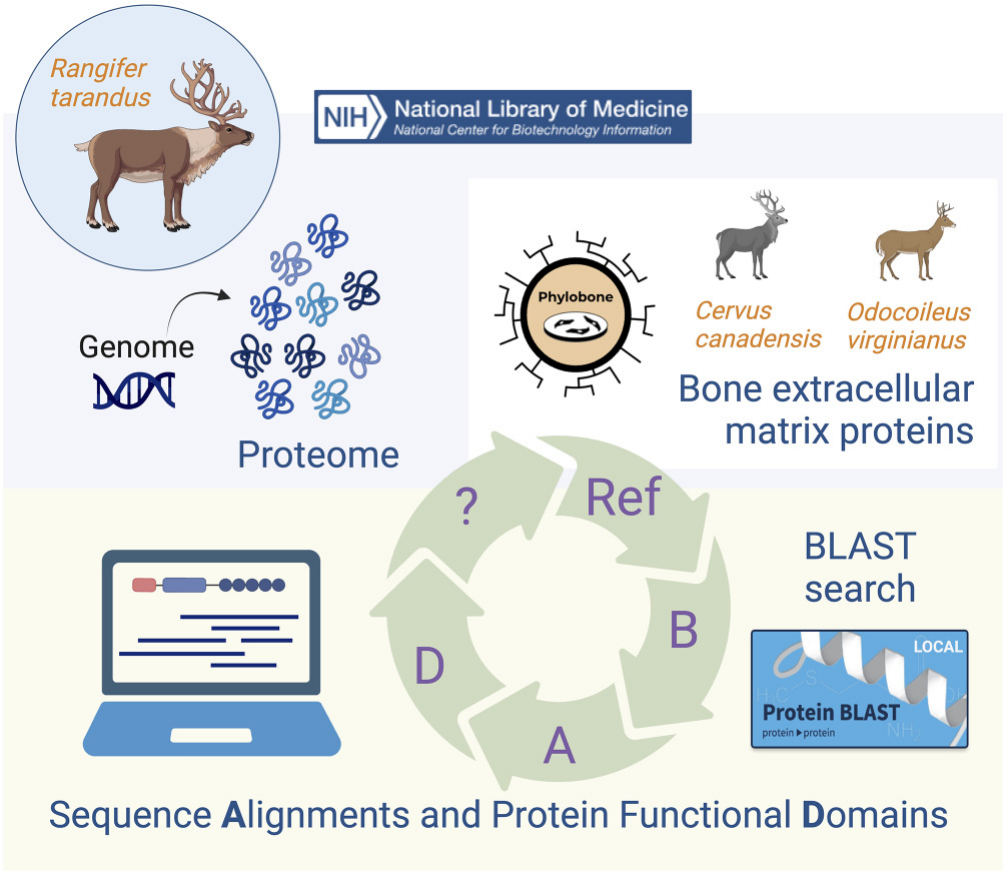
Workflow for the annotation of bone extracellular matrix (ECM) proteins. The reindeer (*Rangifer tarandus*) proteome was obtained from the NCBI's Assembly database. Each protein was mapped onto reference proteins from the Phylobone database. Putative bone ECM proteins were identified with a local BLAST (B)^9^ search, aligned (A)^10^ with elk (*C. canadensis*) or white-tailed deer (*Odocoileus virginianus texanus*) sequences, and annotated for protein functional domains (D).^11^

Here, we used a chromosome-level assembly of a reindeer (*Rangifer tarandus*) genome^5^ as an example of how to utilize the Phylobone database^4^ to annotate bone ECM proteins in novel proteomes. The initial Phylobone database included the annotation of six members of the Cervidae family (including *Cervus hanglu yarkandensis*, *Odocoileus virginianus texanus*, *Cervus canadensis*, *Cervus elaphus*, *Muntiacus muntjak* and *Muntiacus reevesi*), as they have been suggested as potential models for bone regeneration.^4^ Although, the reindeer genome has been fully sequenced at a chromosome level,^5–7^ it was not included in the first version of the database due to the lack of annotation of several proteins. Extracting proteins from the mineral-rich bone ECM is challenging, requiring decalcification and chemical treatments for analysis.^8^ Bioinformatics resources, such as the Phylobone database,^4^ and the workflow (described here) for the annotation of bone ECM proteins will be useful in future studies (Figure 1).

## Material and methods

### Protein sequences

We collected 26,502 protein-coding sequences of *R. tarandus platyrhyncus* (a subspecies of reindeer commonly referred to as Svalbard reindeer) from the National Center for Biotechnology Information's Assembly database (GCA_951394145.1).^5^

### Basic local alignment search tool (BLAST)

A BLAST^9^ search was performed, using bone ECM proteins of *C. canadensis* from Phylobone^4^ as a query, to identify putative bone ECM proteins in the *R. tarandus platyrhyncus* proteome. The BLAST search was performed locally in a cluster computer of the Finnish IT Center for Science (CSC) using the commands *makeblastdb* and *blasp*. Each best BLAST hit (e-value: 10^−6^) was further analyzed for the final annotation of bone ECM proteins.

### Pairwise protein alignments

Each putative ECM protein from the *R. tarandus platyrhyncus* proteome was aligned with an orthologous sequence from elk (*C. canadensis*) using the program Muscle.^10^ Elk was used as a reference species because there are 245 (out of 255) bone ECM protein families predicted in the Phylobone database. In cases where Elk sequences were not available, sequences from white-tailed deer (*O. virginianus texanus*) were used as a reference.

### Identification of protein functional domains

The CD-Search tool (with default parameters)^11^ was used for the annotation of protein functional domains in the set of putative ECM proteins.

### Phylobone database

The final set of putative bone ECM proteins in *R. tarandus* is available at https://phylobone.com. The current version of the database includes seven members of the Cervidae family.

## Results and discussion

We identified a total of 215 sequences of bone ECM proteins in *R. tarandus* that correspond with the 255 protein groups of the Phylobone database (Supplementary table ST1).^4^ We also identified a total of 322 family and superfamily domains present in these bone ECM proteins. These domains include collagen and leucine-rich repeats (LRR), which are the most common domains in the Phylobone database.^4^ Both collagen and LRR are abundant in the bone ECM and are involved in the maintenance of bone homeostasis.^1^ The availability of these sequences may be important for understanding bone regeneration,^4^ as reindeer are capable of developing antlers in male and female individuals.^12^ These data highlight the importance of further research on reindeer biology and genetics to gain a better understanding of bone (and antler) formation and resorption in these animals. It also demonstrates the capacity of Phylobone to be used as a tool for the pre-annotation of new proteomes.

**Figure 2.**
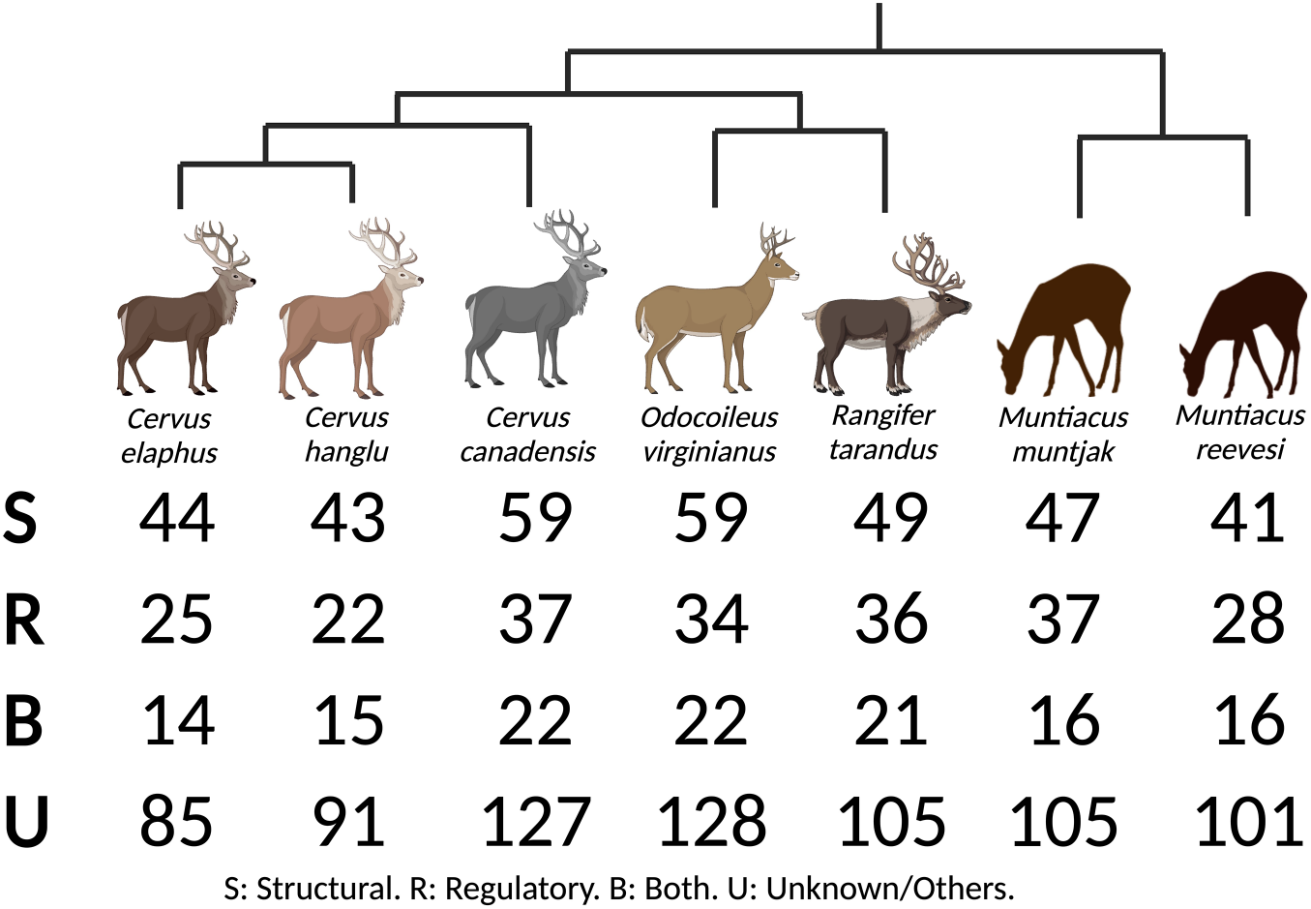
Comparison of bone extracellular matrix (ECM) proteins in the Cervidae family. The Phylobone database contains bone ECM proteins from seven species of the Cervidae family, including *Cervus hanglu yarkandensis*, *Odocoileus virginianus texanus*, *Cervus canadensis*, *Cervus elaphus*, *Muntiacus muntjak*, *Muntiacus reevesi* and *Rangifer tarandus*. Bone ECM proteins include regulatory, structural and unclassified proteins.

Bone ECM proteins of *R. tarandus* have been added to the Phylobone database. Thus, the database contains information about seven deer species in three subfamilies: Cervinae (*C. hanglu yarkandensis*, *C. canadensis* and *C. elaphus*), Odocoileinae (*R. tarandus* and *O. virginianus texanus*) and Muntiacinae (*M. muntjak* and *M. reevesi*). The study of these organisms may shed some light on osteoporosis and bone development due to the rapid growth of their antlers.^13,14^ Each of these species has different levels of annotation and, consequently, different amounts of bone ECM proteins available. For this reason, we were able to retrieve variable amounts of putative bone ECM proteins for Cervidae species (Figure 2). These proteins are either involved in structural and/or regulatory roles or remain unclassified. Some of the unclassified proteins are worth further investigation to disentangle their functional or structural roles in the bone matrix. Pairwise protein alignments of *R. tarandus* with *C. canadensis* and *O. virginianus* show a high conservation identity. We speculate that this is indicative of the presence of evolutionarily conserved elements that may be involved in the annual renewal cycle of deer antlers.^15^

## Conclusions

The workflow for identifying 215 putative bone ECM proteins in reindeer validates the reliability of Phylobone as a resource for pre-annotations of bone ECM proteins in novel organisms. The inclusion of reindeer, along with six other members of the Cervidae family, in the Phylobone database increases the comprehensiveness of this resource and offers an opportunity for future studies to explore the molecular mechanism involved in the regeneration cycle of deer antlers.

## Supplemental Material

sj-docx-1-sci-10.1177_00368504241244666 - Supplemental material for Use of the Phylobone database for the annotation of bone extracellular matrix proteins in reindeer (*Rangifer tarandus*) Supplemental material, sj-docx-1-sci-10.1177_00368504241244666 for Use of the Phylobone database for the annotation of bone extracellular matrix proteins in reindeer (*Rangifer tarandus*) by Alba Sánchez-Reverté, Margalida Fontcuberta-Rigo, Miho Nakamura and Pere Puigbò in Science Progress
