# Supplementary material for: Use of the Phylobone database for the annotation of bone extracellular matrix proteins in reindeer (Rangifer tarandus)
Source: Sci Prog. 2024 Apr 13;107(2):00368504241244666. doi: 10.1177/00368504241244666 (PMC11024589; doi:10.1177/00368504241244666)
Supplement: sj-docx-1-sci-10.1177_00368504241244666 - Supplemental material for Use of the Phylobone database for the annotation of bone extracellular matrix proteins in reindeer (Rangifer tarandus) [file sj-docx-1-sci-10.1177_00368504241244666.docx]

***Current commentary***

Use of the Phylobone database for the annotation of bone extracellular matrix proteins in reindeer (*Rangifer tarandus*)

Alba Sánchez-Reverté^1,&^, Margalida Fontcuberta-Rigo^1,2,&^, Miho Nakamura^1,3,4,^*, and Pere Puigbò^2,5,6,^*

^1^ Medicity Research Laboratory, Faculty of Medicine, University of Turku, Tykistökatu 6, 20520 Turku, Finland

^2^ Department of Biochemistry and Biotechnology, University Rovira i Virgili, 43007 Tarragona, Catalonia, Spain

^3^ Institute of Biomaterials and Bioengineering, Tokyo Medical and Dental University, 2-3-10 Kanda-Surugadai, Chiyoda, Tokyo 1010062 Japan

^4^ Graduate School of Engineering, Tohoku University, 6-6 Aramaki Aza Aoba, Aoba-ku, Sendai, Miyagi 9808579 Japan

^5^ Department of Biology, University of Turku, 20500 Turku, Finland

^6^ Eurecat, Technology Center of Catalonia. Nutrition and Health Unit, Reus, 43204, Catalonia, Spain

^&^ Co-first authors listed alphabetically by first name

* Co-senior authors and for correspondence: [miho.nakamura@utu.fi](mailto:miho.nakamura@utu.fi) (M.N.), [pepuav@utu.fi](mailto:pepuav@utu.fi) (P.P.)

**SUPPLEMENTARY TABLES**

**Supplementary table ST1.** List of putative bone ECM proteins in *Rangifer tarandus platyrhyncus*

| **Protein** | **Link** | **Name** | **Domains** | **Phylobone code** |
| --- | --- | --- | --- | --- |
| **CAI9709816.1** | [NCBI](https://www.ncbi.nlm.nih.gov/protein/CAI9709816.1) | **AHSG** | Cystatin, CY, PTZ00144 superfamily | **PB0001** |
| **CAI9692317.1** | [NCBI](https://www.ncbi.nlm.nih.gov/protein/CAI9692317.1) | **F2** | Tryp_SPc, KR, Thrombin_light, GLA | **PB0003** |
| **CAI9694143.1** | [NCBI](https://www.ncbi.nlm.nih.gov/protein/CAI9694143.1) | **MGP** | Gla superfamily | **PB0004** |
| **CAI9705789.1** | [NCBI](https://www.ncbi.nlm.nih.gov/protein/CAI9705789.1) | **BGN\|ASPN** | PRK15370 superfamily, LRRNT | **PB0005\|PB0185** |
| **CAI9689047.1** | [NCBI](https://www.ncbi.nlm.nih.gov/protein/CAI9689047.1) | **SPARC** | EFh_SPARC_like, FSL_SPARC | **PB0006** |
| **CAI9693821.1** | [NCBI](https://www.ncbi.nlm.nih.gov/protein/CAI9693821.1) | **CHAD** | PPP1R42 superfamily, LRR_8, LRR superfamily, LRRCT, LRRNT, PLN00113 superfamily, LRRCT | **PB0007** |
| **CAI9709770.1** | [NCBI](https://www.ncbi.nlm.nih.gov/protein/CAI9709770.1) | **CHAD** | PPP1R42 superfamily, LRR_8, LRR superfamily, LRRCT, LRRNT, PLN00113 superfamily, LRRCT | **PB0007** |
| **CAI9701338.1** | [NCBI](https://www.ncbi.nlm.nih.gov/protein/CAI9701338.1) | **SERPINF1** | serpinF1_PEDF | **PB0009** |
| **CAI9697667.1** | [NCBI](https://www.ncbi.nlm.nih.gov/protein/CAI9697667.1) | **FTL** | Ferritin | **PB0010** |
| **CAI9693990.1** | [NCBI](https://www.ncbi.nlm.nih.gov/protein/CAI9693990.1) | **GAPDH** | PLN02272 superfamily | **PB0011** |
| **CAI9691287.1** | [NCBI](https://www.ncbi.nlm.nih.gov/protein/CAI9691287.1) | **S100A9** | calgranulins | **PB0012** |
| **CAI9691288.1** | [NCBI](https://www.ncbi.nlm.nih.gov/protein/CAI9691288.1) | **S100A9** | calgranulins | **PB0012** |
| **CAI9696359.1** | [NCBI](https://www.ncbi.nlm.nih.gov/protein/CAI9696359.1) | **CTSG** | Tryp_SPc | **PB0013** |
| **CAI9688764.1** | [NCBI](https://www.ncbi.nlm.nih.gov/protein/CAI9688764.1) | **PRTN3** | Tryp_SPc | **PB0014** |
| **CAI9688570.1** | [NCBI](https://www.ncbi.nlm.nih.gov/protein/CAI9688570.1) | **LOX** | Lysyl_oxidase | **PB0015** |
| **CAI9708431.1** | [NCBI](https://www.ncbi.nlm.nih.gov/protein/CAI9708431.1) | **HBA1** | Hb-alpha-like | **PB0017** |
| **CAI9699508.1** | [NCBI](https://www.ncbi.nlm.nih.gov/protein/CAI9699508.1) | **COL1A2** | COLFI, gly_rich_SclB superfamily | **PB0018** |
| **CAI9700981.1** | [NCBI](https://www.ncbi.nlm.nih.gov/protein/CAI9700981.1) | **COL1A1\|COL9A2** | COLFI, gly_rich_SclB superfamily, VWC, Collagen | **PB0019** |
| **CAI9703355.1** | [NCBI](https://www.ncbi.nlm.nih.gov/protein/CAI9703355.1) | **COL1A1\|COL2A1\|COL9A2** | COLFI, gly_rich_SclB superfamily, VWC, Collagen | **PB0019\|PB0129** |
| **CAI9710963.1** | [NCBI](https://www.ncbi.nlm.nih.gov/protein/CAI9710963.1) | **ADPGK** | ADPGK_ADPPFK | **PB0020** |

| **CAI9705646.1** | [NCBI](https://www.ncbi.nlm.nih.gov/protein/CAI9705646.1) | **LOXL2** | Lysyl_oxidase, SR | **PB0022** |
| --- | --- | --- | --- | --- |
| **CAI9707403.1** | [NCBI](https://www.ncbi.nlm.nih.gov/protein/CAI9707403.1) | **BMP3** | TGF_beta_BMP3, TGFb_propeptide superfamily | **PB0023** |
| **CAI9696228.1** | [NCBI](https://www.ncbi.nlm.nih.gov/protein/CAI9696228.1) | **EFEMP2** | EGF_CA, cEGF, FXa_inhibition, vWFA superfamily | **PB0024** |
| **CAI9705075.1** | [NCBI](https://www.ncbi.nlm.nih.gov/protein/CAI9705075.1) | **EFEMP2** | EGF_CA, cEGF, FXa_inhibition, vWFA superfamily | **PB0024** |
| **CAI9702644.1** | [NCBI](https://www.ncbi.nlm.nih.gov/protein/CAI9702644.1) | **FN1** | FN2, FN3, FN1 | **PB0025** |
| **CAI9696571.1** | [NCBI](https://www.ncbi.nlm.nih.gov/protein/CAI9696571.1) | **MFGE8** | FA58C, FA58C, EGF | **PB0026** |
| **CAI9690948.1** | [NCBI](https://www.ncbi.nlm.nih.gov/protein/CAI9690948.1) | **COL11A1** | COLFI, TSPN, gly_rich_SclB superfamily | **PB0027** |
| **CAI9694834.1** | [NCBI](https://www.ncbi.nlm.nih.gov/protein/CAI9694834.1) | **SFRP1** | CRD_SFRP2, NTR_Sfrp1_like, CRD_SFRP1 | **PB0028** |
| **CAI9707757.1** | [NCBI](https://www.ncbi.nlm.nih.gov/protein/CAI9707757.1) | **SFRP1** | CRD_SFRP2, NTR_Sfrp1_like, CRD_SFRP1 | **PB0028** |
| **CAI9688369.1** | [NCBI](https://www.ncbi.nlm.nih.gov/protein/CAI9688369.1) | **C3** | complement_C3_C4_C5, NTR_complement_C3, A2M_N_2, MG4, A2M_recep, A2M, MG1, ANATO, A2M_N, MG3 | **PB0030** |
| **CAI9702854.1** | [NCBI](https://www.ncbi.nlm.nih.gov/protein/CAI9702854.1) | **MATN1** | vWA_Matrilin | **PB0031** |
| **CAI9692283.1** | [NCBI](https://www.ncbi.nlm.nih.gov/protein/CAI9692283.1) | **HSD17B12** | 17beta-HSD1_like_SDR_c, NADB_Rossmann superfamily | **PB0032** |
| **CAI9698451.1** | [NCBI](https://www.ncbi.nlm.nih.gov/protein/CAI9698451.1) | **ANXA2\|ANXA1** | Annexin | **PB0033\|PB0059** |
| **CAI9714309.1** | [NCBI](https://www.ncbi.nlm.nih.gov/protein/CAI9714309.1) | **CFHR2** | PHA02927 superfamily, CCP | **PB0034** |
| **CAI9693563.1** | [NCBI](https://www.ncbi.nlm.nih.gov/protein/CAI9693563.1) | **PROC** | Tryp_SPc, GLA, FXa_inhibition, EGF_CA | **PB0035** |
| **CAI9711334.1** | [NCBI](https://www.ncbi.nlm.nih.gov/protein/CAI9711334.1) | **HTRA1** | degP_htrA_DO superfamily, IB, KAZAL | **PB0036** |
| **CAI9706175.1** | [NCBI](https://www.ncbi.nlm.nih.gov/protein/CAI9706175.1) | **ENPP1** | Lysyl_oxidase, SR, Phosphodiest, NUC, SO | **PB0037** |
| **CAI9708061.1** | [NCBI](https://www.ncbi.nlm.nih.gov/protein/CAI9708061.1) | **ENPP1** | Lysyl_oxidase, SR, Phosphodiest, NUC, SO | **PB0037** |
| **CAI9702654.1** | [NCBI](https://www.ncbi.nlm.nih.gov/protein/CAI9702654.1) | **IGFBP5** | IB, Thyroglobulin_1 | **PB0038** |
| **CAI9712543.1** | [NCBI](https://www.ncbi.nlm.nih.gov/protein/CAI9712543.1) | **CNMD** | BRICHOS | **PB0039** |
| **CAI9703759.1** | [NCBI](https://www.ncbi.nlm.nih.gov/protein/CAI9703759.1) | **COL8A1** | C1Q, gly_rich_SclB superfamily | **PB0040** |
| **CAI9699512.1** | [NCBI](https://www.ncbi.nlm.nih.gov/protein/CAI9699512.1) | **TFPI2** | Kunitz_TFPI2_1-like, Kunitz_TFPI1_TFPI2_3-like, Kunitz-type superfamily | **PB0042** |
| **CAI9714144.1** | [NCBI](https://www.ncbi.nlm.nih.gov/protein/CAI9714144.1) | **TNN** | FReD, FN3, EGF_Tenascin superfamily, EGF_2 | **PB0043** |
| **CAI9712113.1** | [NCBI](https://www.ncbi.nlm.nih.gov/protein/CAI9712113.1) | **COL4A5\|COL4A1** | C4, gly_rich_SclB superfamily | **PB0044\|PB0113** |
| **CAI9714178.1** | [NCBI](https://www.ncbi.nlm.nih.gov/protein/CAI9714178.1) | **QSOX1** | QSOX_Trx1 superfamily, PDI_a_QSOX, FAD_SOX, Evr1_Alr | **PB0045** |
| **CAI9691514.1** | [NCBI](https://www.ncbi.nlm.nih.gov/protein/CAI9691514.1) | **APOA2** | ApoA-II | **PB0047** |
| **CAI9691733.1** | [NCBI](https://www.ncbi.nlm.nih.gov/protein/CAI9691733.1) | **APOA4** | Apolipoprotein | **PB0049** |
| **CAI9708845.1** | [NCBI](https://www.ncbi.nlm.nih.gov/protein/CAI9708845.1) | **PCOLCE** | NTR_PCOLCE, CUB | **PB0050** |
| **CAI9701943.1** | [NCBI](https://www.ncbi.nlm.nih.gov/protein/CAI9701943.1) | **COL12A1** | vWA_collagen_alphaI-XII-like, TSPN, FN3, gly_rich_SclB superfamily | **PB0051** |
| **CAI9709604.1** | [NCBI](https://www.ncbi.nlm.nih.gov/protein/CAI9709604.1) | **CCDC80** | DUF4174, PHA03418 superfamily | **PB0052** |
| **CAI9700065.1** | [NCBI](https://www.ncbi.nlm.nih.gov/protein/CAI9700065.1) | **LAMB2** | Laminin_N, cc_LAMB2_C, Smc superfamily, EGF_Lam, Laminin_EGF | **PB0053** |
| **CAI9701984.1** | [NCBI](https://www.ncbi.nlm.nih.gov/protein/CAI9701984.1) | **COL9A1\|COL9A2** | TSPN, gly_rich_SclB superfamily | **PB0054\|PB0186** |
| **CAI9710291.1** | [NCBI](https://www.ncbi.nlm.nih.gov/protein/CAI9710291.1) | **ACTN1** | CH_ACTN_rpt2, CH_ACTN_rpt1, EFhand_Ca_insen, SPEC, EFh, Spectrin | **PB0055** |
| **CAI9698191.1** | [NCBI](https://www.ncbi.nlm.nih.gov/protein/CAI9698191.1) | **SOD3** | Sod_Cu | **PB0056** |
| **CAI9698702.1** | [NCBI](https://www.ncbi.nlm.nih.gov/protein/CAI9698702.1) | **HMGB2** | HMG-box_HMGB_rpt2, HMG-box_HMGB_rpt1 | **PB0057** |
| **CAI9712390.1** | [NCBI](https://www.ncbi.nlm.nih.gov/protein/CAI9712390.1) | **HMGB2** | HMG-box_HMGB_rpt2, HMG-box_HMGB_rpt1 | **PB0057** |
| **CAI9700134.1** | [NCBI](https://www.ncbi.nlm.nih.gov/protein/CAI9700134.1) | **TF** | PBP2_transferrin_C, Transferrin | **PB0058** |
| **CAI9709504.1** | [NCBI](https://www.ncbi.nlm.nih.gov/protein/CAI9709504.1) | **TF** | PBP2_transferrin_C, Transferrin | **PB0058** |
| **CAI9708313.1** | [NCBI](https://www.ncbi.nlm.nih.gov/protein/CAI9708313.1) | **RNASET2** | Ribonuclease_T2 | **PB0060** |
| **CAI9704445.1** | [NCBI](https://www.ncbi.nlm.nih.gov/protein/CAI9704445.1) | **TNXB** | Fibrinogen_C, FN3, EGF_Tenascin superfamily, EGF_2 | **PB0063** |
| **CAI9696772.1** | [NCBI](https://www.ncbi.nlm.nih.gov/protein/CAI9696772.1) | **CLEC3B** | CLECT_tetranectin_like | **PB0064** |
| **CAI9700153.1** | [NCBI](https://www.ncbi.nlm.nih.gov/protein/CAI9700153.1) | **CLEC3B** | CLECT_tetranectin_like | **PB0064** |
| **CAI9708353.1** | [NCBI](https://www.ncbi.nlm.nih.gov/protein/CAI9708353.1) | **THBS2** | TSP_C, TSPN, VWC, TSP1, TSP_1, TSP_3, EGF_3 | **PB0065** |
| **CAI9709753.1** | [NCBI](https://www.ncbi.nlm.nih.gov/protein/CAI9709753.1) | **MELTF** | Transferrin superfamily, TR_FER | **PB0066** |
| **CAI9712063.1** | [NCBI](https://www.ncbi.nlm.nih.gov/protein/CAI9712063.1) | **F9\|F10** | Tryp_SPc, GLA, FXa_inhibition, EGF_CA | **PB0067\|PB0124** |
| **CAI9696573.1** | [NCBI](https://www.ncbi.nlm.nih.gov/protein/CAI9696573.1) | **ACAN** | CLECT_CSPGs, Ig_Aggrecan, Link_domain_CSPGs_modules_1_3, Link_domain_CSPGs_modules_2_4, , CCP, EGF_CA, PHA03307 superfamily, PRK15387 superfamily | **PB0068** |
| **CAI9712430.1** | [NCBI](https://www.ncbi.nlm.nih.gov/protein/CAI9712430.1) | **POSTN** | Fasciclin | **PB0069** |
| **CAI9710580.1** | [NCBI](https://www.ncbi.nlm.nih.gov/protein/CAI9710580.1) | **NID2** | nidG2, TY, LY, EGF_3, EGF_CA | **PB0070** |
| **CAI9699132.1** | [NCBI](https://www.ncbi.nlm.nih.gov/protein/CAI9699132.1) | **AEBP1** | Peptidase_M14_like superfamily, FA58C, Peptidase_M14NE-CP-C_like | **PB0072** |

| **CAI9712009.1** | [NCBI](https://www.ncbi.nlm.nih.gov/protein/CAI9712009.1) | **NID1** | nidG2, NIDO, Thyroglobulin_1, LY, EGF_3, FXa_inhibition, Ldl_recept_b, EGF_CA, LY | **PB0075** |
| --- | --- | --- | --- | --- |
| **CAI9704793.1** | [NCBI](https://www.ncbi.nlm.nih.gov/protein/CAI9704793.1) | **COL21A1** | vWFA superfamily, gly_rich_SclB superfamily | **PB0076** |
| **CAI9706696.1** | [NCBI](https://www.ncbi.nlm.nih.gov/protein/CAI9706696.1) | **APOB** | Vitellogenin_N superfamily, DUF1943, DUF1081, ApoB100_C, 235kDa-fam superfamily | **PB0077** |
| **CAI9689163.1** | [NCBI](https://www.ncbi.nlm.nih.gov/protein/CAI9689163.1) | **HAPLN1** | Ig_LP_like, Link_domain_HAPLN_module_1 | **PB0078** |
| **CAI9688241.1** | [NCBI](https://www.ncbi.nlm.nih.gov/protein/CAI9688241.1) | **COL5A3** | COLFI, LamG superfamily, gly_rich_SclB superfamily | **PB0079** |
| **CAI9705974.1** | [NCBI](https://www.ncbi.nlm.nih.gov/protein/CAI9705974.1) | **C5** | A2M_comp, NTR_like superfamily, A2M_N_2, A2M_recep, A2M, MG1, MG4, ANATO, A2M_N, MG3 superfamily | **PB0080** |
| **CAI9709385.1** | [NCBI](https://www.ncbi.nlm.nih.gov/protein/CAI9709385.1) | **COL6A2** | vWA_collagen_alpha_1-VI-type, vWFA superfamily, gly_rich_SclB superfamily, VWA | **PB0081** |
| **CAI9690650.1** | [NCBI](https://www.ncbi.nlm.nih.gov/protein/CAI9690650.1) | **PODN** | PLN00113 superfamily, LRRNT | **PB0082** |
| **CAI9693536.1** | [NCBI](https://www.ncbi.nlm.nih.gov/protein/CAI9693536.1) | **COL5A2** | COLFI, gly_rich_SclB superfamily, VWC, Collagen | **PB0084** |
| **CAI9709815.1** | [NCBI](https://www.ncbi.nlm.nih.gov/protein/CAI9709815.1) | **FETUB** | CY, CY superfamily | **PB0085** |
| **CAI9691734.1** | [NCBI](https://www.ncbi.nlm.nih.gov/protein/CAI9691734.1) | **APOA1** | Apolipoprotein | **PB0086** |
| **CAI9697534.1** | [NCBI](https://www.ncbi.nlm.nih.gov/protein/CAI9697534.1) | **APOC2** | Apo-CII | **PB0088** |
| **CAI9690335.1** | [NCBI](https://www.ncbi.nlm.nih.gov/protein/CAI9690335.1) | **spp2** | Spp-24 | **PB0089** |
| **CAI9690309.1** | [NCBI](https://www.ncbi.nlm.nih.gov/protein/CAI9690309.1) | **COL6A3** | vWA_collagen_alpha3-VI-like, vWFA superfamily, vWA_collagen, gly_rich_SclB superfamily, VWA, Kunitz_collagen_alpha3_VI, , FN3, Collagen, HC2 superfamily | **PB0090** |
| **CAI9702841.1** | [NCBI](https://www.ncbi.nlm.nih.gov/protein/CAI9702841.1) | **COL16A1** | TSPN, gly_rich_SclB superfamily | **PB0091** |
| **CAI9703025.1** | [NCBI](https://www.ncbi.nlm.nih.gov/protein/CAI9703025.1) | **ALPL** | Alk_phosphatase | **PB0092** |
| **CAI9713530.1** | [NCBI](https://www.ncbi.nlm.nih.gov/protein/CAI9713530.1) | **ITIH6** | ITI_HC_C, vWA_interalpha_trypsin_inhibitor | **PB0093** |
| **CAI9687954.1** | [NCBI](https://www.ncbi.nlm.nih.gov/protein/CAI9687954.1) | **CILP2** | Mucin2_WxxW, Ig_3, TSP1, CarboxypepD_reg | **PB0094** |
| **CAI9712062.1** | [NCBI](https://www.ncbi.nlm.nih.gov/protein/CAI9712062.1) | **PROZ** | Trypsin, GLA, FXa_inhibition, EGF_CA | **PB0095** |
| **CAI9709388.1** | [NCBI](https://www.ncbi.nlm.nih.gov/protein/CAI9709388.1) | **COL6A1** | vWA_collagen_alpha_1-VI-type, gly_rich_SclB superfamily | **PB0096** |
| **CAI9713631.1** | [NCBI](https://www.ncbi.nlm.nih.gov/protein/CAI9713631.1) | **FMOD** | PPP1R42 superfamily | **PB0098** |
| **CAI9711039.1** | [NCBI](https://www.ncbi.nlm.nih.gov/protein/CAI9711039.1) | **CILP** | Mucin2_WxxW, Ig_3, TSP1, CarboxypepD_reg | **PB0099** |
| **CAI9691210.1** | [NCBI](https://www.ncbi.nlm.nih.gov/protein/CAI9691210.1) | **CTSK** | Peptidase_C1, Inhibitor_I29 | **PB0100** |
| **CAI9698165.1** | [NCBI](https://www.ncbi.nlm.nih.gov/protein/CAI9698165.1) | **SPP1** | OSTEO | **PB0102** |
| **CAI9700622.1** | [NCBI](https://www.ncbi.nlm.nih.gov/protein/CAI9700622.1) | **METRNL** | #N/A | **PB0103** |
| **CAI9699331.1** | [NCBI](https://www.ncbi.nlm.nih.gov/protein/CAI9699331.1) | **LAMB4\|LAMB1** | Laminin_N, cc_LAMB1_C, Smc superfamily, EGF_Lam, Laminin_EGF | **PB0105\|PB0128** |
| **CAI9688340.1** | [NCBI](https://www.ncbi.nlm.nih.gov/protein/CAI9688340.1) | **CCL25** | IL8 | **PB0106** |
| **CAI9712746.1** | [NCBI](https://www.ncbi.nlm.nih.gov/protein/CAI9712746.1) | **MMP9** | HX, Peptidase_M10, FN2 | **PB0107** |
| **CAI9710693.1** | [NCBI](https://www.ncbi.nlm.nih.gov/protein/CAI9710693.1) | **THBS1\|BMP1** | TSP_C, TSPN, TSP1, VWC, TSP_3 | **PB0108** |
| **CAI9704409.1** | [NCBI](https://www.ncbi.nlm.nih.gov/protein/CAI9704409.1) | **APOM** | Lipocalin superfamily | **PB0109** |
| **CAI9712064.1** | [NCBI](https://www.ncbi.nlm.nih.gov/protein/CAI9712064.1) | **F7** | Tryp_SPc, GLA, FXa_inhibition, EGF_CA | **PB0110** |
| **CAI9705092.1** | [NCBI](https://www.ncbi.nlm.nih.gov/protein/CAI9705092.1) | **LTBP3** | TB, EGF_CA, PHA03247 superfamily, TB superfamily | **PB0111** |
| **CAI9691602.1** | [NCBI](https://www.ncbi.nlm.nih.gov/protein/CAI9691602.1) | **MMP13** | Peptidase_M10, HX, PG_binding_1 | **PB0112** |
| **CAI9712048.1** | [NCBI](https://www.ncbi.nlm.nih.gov/protein/CAI9712048.1) | **GAS6** | LamG, GLA, EGF_CA, FXa_inhibition | **PB0114** |
| **CAI9707871.1** | [NCBI](https://www.ncbi.nlm.nih.gov/protein/CAI9707871.1) | **PDGFRL** | Ig superfamily | **PB0115** |
| **CAI9701418.1** | [NCBI](https://www.ncbi.nlm.nih.gov/protein/CAI9701418.1) | **VTN** | HX, SO | **PB0116** |
| **CAI9704435.1** | [NCBI](https://www.ncbi.nlm.nih.gov/protein/CAI9704435.1) | **C2** | vWFA superfamily, Tryp_SPc, CCP, PHA02927 superfamily | **PB0118** |
| **CAI9711552.1** | [NCBI](https://www.ncbi.nlm.nih.gov/protein/CAI9711552.1) | **SFRP5** | CRD_SFRP5, NTR_Sfrp1_like | **PB0119** |
| **CAI9703803.1** | [NCBI](https://www.ncbi.nlm.nih.gov/protein/CAI9703803.1) | **PROS1** | Laminin_G_1, GLA, FXa_inhibition, Laminin_G_2, EGF_CA, cEGF, EGF | **PB0120** |
| **CAI9691352.1** | [NCBI](https://www.ncbi.nlm.nih.gov/protein/CAI9691352.1) | **THBS3** | TSP_C, LamG superfamily, TSP-3cc, TSP_3, EGF_CA, MSCRAMM_ClfA superfamily, EGF_3 | **PB0122** |
| **CAI9713093.1** | [NCBI](https://www.ncbi.nlm.nih.gov/protein/CAI9713093.1) | **LAMA5** | Laminin_N, Laminin_I superfamily, Laminin_B, Laminin_II superfamily, LamG, Laminin_EGF, EGF_Lam, Smc superfamily | **PB0123** |
| **CAI9697664.1** | [NCBI](https://www.ncbi.nlm.nih.gov/protein/CAI9697664.1) | **NUCB1** | EF-hand_7, DUF5401 superfamily | **PB0125** |
| **CAI9705787.1** | [NCBI](https://www.ncbi.nlm.nih.gov/protein/CAI9705787.1) | **OGN** | LRR_8 | **PB0127** |
| **CAI9698047.1** | [NCBI](https://www.ncbi.nlm.nih.gov/protein/CAI9698047.1) | **NDNF** | NDNF_C, DUF2369 | **PB0130** |
| **CAI9698179.1** | [NCBI](https://www.ncbi.nlm.nih.gov/protein/CAI9698179.1) | **SLIT2\|SLIT3** | LamG, LRR_8, PCC superfamily, EGF_CA, PCC superfamily, LRRNT | **PB0131\|PB0168** |
| **CAI9708867.1** | [NCBI](https://www.ncbi.nlm.nih.gov/protein/CAI9708867.1) | **SERPINE1** | serpinE1_PAI-1 | **PB0132** |
| **CAI9702185.1** | [NCBI](https://www.ncbi.nlm.nih.gov/protein/CAI9702185.1) | **LAMA3** | LamNT, Laminin_I superfamily, Laminin_II, Laminin_B, LamG, Laminin_EGF, EGF_Lam, SMC_prok_B superfamily | **PB0135** |
| **CAI9711406.1** | [NCBI](https://www.ncbi.nlm.nih.gov/protein/CAI9711406.1) | **HABP2** | Tryp_SPc, KR, EGF | **PB0136** |

| **CAI9713633.1** | [NCBI](https://www.ncbi.nlm.nih.gov/protein/CAI9713633.1) | **PRELP** | PRK15370 superfamily, LRR_8, LRRNT | **PB0138** |
| --- | --- | --- | --- | --- |
| **CAI9712112.1** | [NCBI](https://www.ncbi.nlm.nih.gov/protein/CAI9712112.1) | **COL4A6\|COL4A2** | C4, gly_rich_SclB superfamily | **PB0140\|PB0147** |
| **CAI9701816.1** | [NCBI](https://www.ncbi.nlm.nih.gov/protein/CAI9701816.1) | **LAMA4** | Laminin_I superfamily, Laminin_II superfamily, LamG, EGF_Lam, Tar | **PB0141** |
| **CAI9714088.1** | [NCBI](https://www.ncbi.nlm.nih.gov/protein/CAI9714088.1) | **AGRN** | PTPc-N11_6, SH2_C-SH2_SHP_like, NtA, Laminin_G_1, SEA, SH2 superfamily, Laminin_EGF, KAZAL, KAZAL_FS, KAZAL_FS superfamily, , EGF_CA, MFS superfamily, EGF | **PB0142** |
| **CAI9699364.1** | [NCBI](https://www.ncbi.nlm.nih.gov/protein/CAI9699364.1) | **LRRC17** | LRR_8, LRR superfamily | **PB0143** |
| **CAI9705951.1** | [NCBI](https://www.ncbi.nlm.nih.gov/protein/CAI9705951.1) | **LRRC17\|TNC** | Fibrinogen_C, FN3, EGF_Tenascin superfamily, EGF_2 | **PB0139** |
| **CAI9713104.1** | [NCBI](https://www.ncbi.nlm.nih.gov/protein/CAI9713104.1) | **COL9A3\|COL9A2** | gly_rich_SclB superfamily, gly_rich_SclB superfamily | **PB0145** |
| **CAI9711059.1** | [NCBI](https://www.ncbi.nlm.nih.gov/protein/CAI9711059.1) | **THBS4** | TSP_C, LamG superfamily, TSP-4cc, TSP_3, EGF_CA, EGF, gliding_CglD superfamily | **PB0146** |
| **CAI9705790.1** | [NCBI](https://www.ncbi.nlm.nih.gov/protein/CAI9705790.1) | **ECM2** | VWC, LRR, LRR_8 | **PB0148** |
| **CAI9691870.1** | [NCBI](https://www.ncbi.nlm.nih.gov/protein/CAI9691870.1) | **SPON1** | Spond_N superfamily, Reeler | **PB0150** |
| **CAI9704762.1** | [NCBI](https://www.ncbi.nlm.nih.gov/protein/CAI9704762.1) | **COL11A2** | COLFI, TSPN, gly_rich_SclB superfamily, Collagen | **PB0151** |
| **CAI9703019.1** | [NCBI](https://www.ncbi.nlm.nih.gov/protein/CAI9703019.1) | **HSPG2** | LamB, Laminin_B, IgI_Perlecan_like, LamG, SEA, Ig superfamily, I-set, IG_like, Ig_3, EGF_Lam, , LDLa, Laminin_EGF, EGF_CA, PLN02983 superfamily | **PB0152** |
| **CAI9708238.1** | [NCBI](https://www.ncbi.nlm.nih.gov/protein/CAI9708238.1) | **FNDC1** | PHA03247 superfamily, FN3, FN3, PTZ00449 superfamily | **PB0154** |
| **CAI9703595.1** | [NCBI](https://www.ncbi.nlm.nih.gov/protein/CAI9703595.1) | **LUM** | PRK15370 superfamily | **PB0155** |
| **CAI9710449.1** | [NCBI](https://www.ncbi.nlm.nih.gov/protein/CAI9710449.1) | **FBN1** | Fibrillin_U_N superfamily, TB, EGF_3, FXa_inhibition, EGF_CA, cEGF, vWFA superfamily | **PB0156** |
| **CAI9709584.1** | [NCBI](https://www.ncbi.nlm.nih.gov/protein/CAI9709584.1) | **PCOLCE2** | NTR_PCOLCE, CUB | **PB0157** |
| **CAI9688245.1** | [NCBI](https://www.ncbi.nlm.nih.gov/protein/CAI9688245.1) | **ANGPTL1** | FReD | **PB0158** |
| **CAI9704436.1** | [NCBI](https://www.ncbi.nlm.nih.gov/protein/CAI9704436.1) | **CFB** | vWA_complement_factors, Tryp_SPc, CCP, PHA02927 superfamily | **PB0159** |
| **CAI9713755.1** | [NCBI](https://www.ncbi.nlm.nih.gov/protein/CAI9713755.1) | **TGFB2** | TGF_beta_TGFB2, TGFb_propeptide superfamily | **PB0160** |
| **CAI9699922.1** | [NCBI](https://www.ncbi.nlm.nih.gov/protein/CAI9699922.1) | **FLNA** | CH_FLNB_rpt1, CH_FLNB_rpt2, IG_FLMN | **PB0161** |
| **CAI9697999.1** | [NCBI](https://www.ncbi.nlm.nih.gov/protein/CAI9697999.1) | **CFI** | Tryp_SPc, FIMAC, SR, Ldl_recept_a, LDLa | **PB0162** |
| **CAI9693199.1** | [NCBI](https://www.ncbi.nlm.nih.gov/protein/CAI9693199.1) | **COL14A1** | vWA_collagen_alphaI-XII-like, TSPN, gly_rich_SclB superfamily, FN3 | **PB0163** |
| **CAI9699257.1** | [NCBI](https://www.ncbi.nlm.nih.gov/protein/CAI9699257.1) | **BMPER** | VWD, C8, TIL, VWC, VWC superfamily | **PB0164** |
| **CAI9701339.1** | [NCBI](https://www.ncbi.nlm.nih.gov/protein/CAI9701339.1) | **SERPINF2** | serpinF2_A2AP | **PB0165** |
| **CAI9707541.1** | [NCBI](https://www.ncbi.nlm.nih.gov/protein/CAI9707541.1) | **CPZ** | M14_CPZ, CRD_FZ superfamily, Peptidase_M14NE-CP-C_like | **PB0167** |
| **CAI9707538.1** | [NCBI](https://www.ncbi.nlm.nih.gov/protein/CAI9707538.1) | **HTRA3** | degP_htrA_DO superfamily, IGFBP, KAZAL | **PB0171** |
| **CAI9695725.1** | [NCBI](https://www.ncbi.nlm.nih.gov/protein/CAI9695725.1) | **ESM1** | IGFBP | **PB0172** |
| **CAI9705627.1** | [NCBI](https://www.ncbi.nlm.nih.gov/protein/CAI9705627.1) | **BMP1** | ZnMc_BMP1_TLD, CUB, FXa_inhibition | **PB0173** |
| **CAI9696195.1** | [NCBI](https://www.ncbi.nlm.nih.gov/protein/CAI9696195.1) | **SERPINA1** | serpinA6_CBG | **PB0175** |
| **CAI9688867.1** | [NCBI](https://www.ncbi.nlm.nih.gov/protein/CAI9688867.1) | **TGFBI** | Fasciclin | **PB0176** |
| **CAI9695369.1** | [NCBI](https://www.ncbi.nlm.nih.gov/protein/CAI9695369.1) | **TCN2** | Cobalamin_bind, DUF4430 superfamily | **PB0177** |
| **CAI9693547.1** | [NCBI](https://www.ncbi.nlm.nih.gov/protein/CAI9693547.1) | **MSTN** | TGF_beta_GDF8, TGFb_propeptide superfamily | **PB0178** |
| **CAI9697532.1** | [NCBI](https://www.ncbi.nlm.nih.gov/protein/CAI9697532.1) | **APOE** | Apolipoprotein | **PB0179** |
| **CAI9699692.1** | [NCBI](https://www.ncbi.nlm.nih.gov/protein/CAI9699692.1) | **CRTAP** | #N/A | **PB0182** |
| **CAI9702844.1** | [NCBI](https://www.ncbi.nlm.nih.gov/protein/CAI9702844.1) | **TINAGL1** | Peptidase_C1A_CathepsinB | **PB0183** |
| **CAI9714206.1** | [NCBI](https://www.ncbi.nlm.nih.gov/protein/CAI9714206.1) | **LAMC1** | LamNT, Laminin_B, SbcC superfamily, Laminin_EGF, EGF_Lam | **PB0184** |
| **CAI9690457.1** | [NCBI](https://www.ncbi.nlm.nih.gov/protein/CAI9690457.1) | **COL9A2** | gly_rich_SclB superfamily | **PB0186** |
| **CAI9709813.1** | [NCBI](https://www.ncbi.nlm.nih.gov/protein/CAI9709813.1) | **KNG1** | Cystatin, CY | **PB0187** |
| **CAI9700524.1** | [NCBI](https://www.ncbi.nlm.nih.gov/protein/CAI9700524.1) | **TIMP2** | NTR_TIMP | **PB0188** |
| **CAI9703286.1** | [NCBI](https://www.ncbi.nlm.nih.gov/protein/CAI9703286.1) | **LYZ** | LYZ_C | **PB0189** |
| **CAI9691062.1** | [NCBI](https://www.ncbi.nlm.nih.gov/protein/CAI9691062.1) | **OLFML3** | OLF, ATG16 superfamily | **PB0190** |
| **CAI9713868.1** | [NCBI](https://www.ncbi.nlm.nih.gov/protein/CAI9713868.1) | **DPT** | DERM | **PB0191** |
| **CAI9713896.1** | [NCBI](https://www.ncbi.nlm.nih.gov/protein/CAI9713896.1) | **MYOC** | OLF superfamily, SMC_prok_A superfamily | **PB0193** |
| **CAI9712786.1** | [NCBI](https://www.ncbi.nlm.nih.gov/protein/CAI9712786.1) | **MATN4** | vWA_Matrilin, vWFA superfamily, FXa_inhibition | **PB0194** |
| **CAI9706710.1** | [NCBI](https://www.ncbi.nlm.nih.gov/protein/CAI9706710.1) | **MATN3** | vWFA superfamily, Matrilin_ccoil, FXa_inhibition | **PB0195** |
| **CAI9708062.1** | [NCBI](https://www.ncbi.nlm.nih.gov/protein/CAI9708062.1) | **CCN2** | TSP1_CCN, GHB_like superfamily, VWC superfamily, IGFBP | **PB0196** |
| **CAI9703596.1** | [NCBI](https://www.ncbi.nlm.nih.gov/protein/CAI9703596.1) | **KERA** | PRK15370 superfamily, LRR | **PB0197** |
| **CAI9705788.1** | [NCBI](https://www.ncbi.nlm.nih.gov/protein/CAI9705788.1) | **KERA** | PRK15370 superfamily, LRR | **PB0197** |
| **CAI9703594.1** | [NCBI](https://www.ncbi.nlm.nih.gov/protein/CAI9703594.1) | **DCN** | PRK15370 superfamily, LRR_8, LRR_8 superfamily | **PB0198** |

| **CAI9696196.1** | [NCBI](https://www.ncbi.nlm.nih.gov/protein/CAI9696196.1) | **SERPINA10** | serpinA10_PZI | **PB0199** |
| --- | --- | --- | --- | --- |
| **CAI9698642.1** | [NCBI](https://www.ncbi.nlm.nih.gov/protein/CAI9698642.1) | **CLU** | Clusterin | **PB0200** |
| **CAI9710202.1** | [NCBI](https://www.ncbi.nlm.nih.gov/protein/CAI9710202.1) | **TGFB3** | TGF_beta_TGFB3, TGFb_propeptide | **PB0201** |
| **CAI9693030.1** | [NCBI](https://www.ncbi.nlm.nih.gov/protein/CAI9693030.1) | **CTHRC1** | Collagen | **PB0202** |
| **CAI9697004.1** | [NCBI](https://www.ncbi.nlm.nih.gov/protein/CAI9697004.1) | **MMP2** | HX, Peptidase_M10, FN2, PG_binding_1 | **PB0204** |
| **CAI9699055.1** | [NCBI](https://www.ncbi.nlm.nih.gov/protein/CAI9699055.1) | **CALU** | EFh_CREC_Calumenin | **PB0205** |
| **CAI9699501.1** | [NCBI](https://www.ncbi.nlm.nih.gov/protein/CAI9699501.1) | **PON2** | Arylesterase, SGL superfamily, Arylesterase | **PB0207** |
| **CAI9699503.1** | [NCBI](https://www.ncbi.nlm.nih.gov/protein/CAI9699503.1) | **PON2** | Arylesterase, SGL superfamily, Arylesterase | **PB0207** |
| **CAI9694829.1** | [NCBI](https://www.ncbi.nlm.nih.gov/protein/CAI9694829.1) | **FGB** | TGF_beta_BMP4_BMP2B, TGFb_propeptide | **PB0208** |
| **CAI9691825.1** | [NCBI](https://www.ncbi.nlm.nih.gov/protein/CAI9691825.1) | **HSPA8** | PTZ00009 superfamily | **PB0209** |
| **CAI9694080.1** | [NCBI](https://www.ncbi.nlm.nih.gov/protein/CAI9694080.1) | **OLR1** | TGF_beta_BMP4_BMP2B, TGFb_propeptide | **PB0210** |
| **CAI9698053.1** | [NCBI](https://www.ncbi.nlm.nih.gov/protein/CAI9698053.1) | **ANXA5** | Annexin | **PB0211** |
| **CAI9711612.1** | [NCBI](https://www.ncbi.nlm.nih.gov/protein/CAI9711612.1) | **RBP4** | lipocalin_RBP_like | **PB0212** |
| **CAI9710715.1** | [NCBI](https://www.ncbi.nlm.nih.gov/protein/CAI9710715.1) | **GREM1v** | DAN | **PB0213** |
| **CAI9694827.1** | [NCBI](https://www.ncbi.nlm.nih.gov/protein/CAI9694827.1) | **FGG** | TGF_beta_BMP4_BMP2B, TGFb_propeptide | **PB0214** |
| **CAI9702736.1** | [NCBI](https://www.ncbi.nlm.nih.gov/protein/CAI9702736.1) | **SERPINE2** | serpinE2_GDN | **PB0215** |
| **CAI9712489.1** | [NCBI](https://www.ncbi.nlm.nih.gov/protein/CAI9712489.1) | **ITM2B** | BRICHOS | **PB0216** |
| **CAI9692310.1** | [NCBI](https://www.ncbi.nlm.nih.gov/protein/CAI9692310.1) | **MDK** | PTN_MK_N superfamily | **PB0218** |
| **CAI9693514.1** | [NCBI](https://www.ncbi.nlm.nih.gov/protein/CAI9693514.1) | **FRZB** | TGF_beta_BMP4_BMP2B, TGFb_propeptide | **PB0220** |
| **CAI9695734.1** | [NCBI](https://www.ncbi.nlm.nih.gov/protein/CAI9695734.1) | **FST** | TGF_beta_BMP4_BMP2B, TGFb_propeptide | **PB0221** |
| **CAI9694057.1** | [NCBI](https://www.ncbi.nlm.nih.gov/protein/CAI9694057.1) | **A2M** | A2M_2, A2M, A2M_N_2, A2M_recep, MG4, MG3, A2M_N | **PB0222** |
| **CAI9703867.1** | [NCBI](https://www.ncbi.nlm.nih.gov/protein/CAI9703867.1) | **APP** | A4_EXTRA, APP_E2, JMTM_Notch_APP superfamily, Kunitz_ABPP-like | **PB0223** |
| **CAI9706487.1** | [NCBI](https://www.ncbi.nlm.nih.gov/protein/CAI9706487.1) | **CAPG** | gelsolin_S1_like, gelsolin_S2_like, gelsolin_S3_like | **PB0224** |
| **CAI9705103.1** | [NCBI](https://www.ncbi.nlm.nih.gov/protein/CAI9705103.1) | **CAPN1** | Peptidase_C2, EFh_PEF_CAPN1, Calpain_III | **PB0225** |
| **CAI9713789.1** | [NCBI](https://www.ncbi.nlm.nih.gov/protein/CAI9713789.1) | **CAPN2** | Peptidase_C2, EFh_PEF_CAPN2, Calpain_III | **PB0226** |
| **CAI9697341.1** | [NCBI](https://www.ncbi.nlm.nih.gov/protein/CAI9697341.1) | **CAPNS1** | EFh_PEF_CPNS1_2 | **PB0227** |
| **CAI9692260.1** | [NCBI](https://www.ncbi.nlm.nih.gov/protein/CAI9692260.1) | **CD44** | Link_domain_CD44_like | **PB0228** |
| **CAI9709393.1** | [NCBI](https://www.ncbi.nlm.nih.gov/protein/CAI9709393.1) | **COL18A1** | Endostatin-like, DUF959 superfamily, CRD_FZ superfamily, LamG superfamily,  gly_rich_SclB superfamily, Collagen_trimer | **PB0229** |
| **CAI9693535.1** | [NCBI](https://www.ncbi.nlm.nih.gov/protein/CAI9693535.1) | **COL3A1** | COLFI, gly_rich_SclB superfamily, VWC, Collagen | **PB0230** |
| **CAI9693738.1** | [NCBI](https://www.ncbi.nlm.nih.gov/protein/CAI9693738.1) | **FBLN1** | ANATO, EGF_CA, FXa_inhibition, cEGF | **PB0231** |
| **CAI9694828.1** | [NCBI](https://www.ncbi.nlm.nih.gov/protein/CAI9694828.1) | **FGA** | FReD, Fib_alpha, Fibrinogen_aC, COG5651 superfamily | **PB0232** |
| **CAI9705980.1** | [NCBI](https://www.ncbi.nlm.nih.gov/protein/CAI9705980.1) | **GSN** | gelsolin_S1_like, gelsolin_S4_like, gelsolin_S6_like, gelsolin_S2_like, gelsolin_S5_like, gelsolin_S3_like | **PB0233** |
| **CAI9703555.1** | [NCBI](https://www.ncbi.nlm.nih.gov/protein/CAI9703555.1) | **ITGA5** | Integrin_alpha2, Int_alpha | **PB0234** |
| **CAI9693527.1** | [NCBI](https://www.ncbi.nlm.nih.gov/protein/CAI9693527.1) | **ITGAV** | Integrin_alpha2, Int_alpha, VCBS | **PB0235** |
| **CAI9713482.1** | [NCBI](https://www.ncbi.nlm.nih.gov/protein/CAI9713482.1) | **ITGB1** | INB, Integrin_B_tail, Integrin_b_cyt, I-EGF_1 | **PB0236** |
| **CAI9712502.1** | [NCBI](https://www.ncbi.nlm.nih.gov/protein/CAI9712502.1) | **LCP1** | CH_PLS_rpt1, CH_SF superfamily, CH_PLS2_rpt2, CH_PLS2_rpt4, EFh | **PB0237** |
| **CAI9708255.1** | [NCBI](https://www.ncbi.nlm.nih.gov/protein/CAI9708255.1) | **PLG** | Tryp_SPc, KR, Kringle, PAN_AP_HGF | **PB0238** |
| **CAI9688196.1** | [NCBI](https://www.ncbi.nlm.nih.gov/protein/CAI9688196.1) | **PRDX4** | PRX_Typ2cys | **PB0239** |
| **CAI9702474.1** | [NCBI](https://www.ncbi.nlm.nih.gov/protein/CAI9702474.1) | **SERPINB5** | serpinB5_maspin | **PB0240** |
| **CAI9695575.1** | [NCBI](https://www.ncbi.nlm.nih.gov/protein/CAI9695575.1) | **SH3PXD2B\|SH3PXD2B** | PX_FISH, SH3_Tks4_1, SH3_Tks4_2, SH3_Tks4_3, SH3 superfamily, PHA03307 superfamily | **PB0241** |
| **CAI9702157.1** | [NCBI](https://www.ncbi.nlm.nih.gov/protein/CAI9702157.1) | **TTR** | TR_THY | **PB0242** |
| **CAI9689162.1** | [NCBI](https://www.ncbi.nlm.nih.gov/protein/CAI9689162.1) | **VCAN** | CLECT_CSPGs, Ig_Versican, Link_domain_CSPGs_modules_2_4, Link_domain_CSPGs_modules_1_3, EGF_CA, CCP, PRK14949 superfamily | **PB0243** |
| **CAI9698907.1** | [NCBI](https://www.ncbi.nlm.nih.gov/protein/CAI9698907.1) | **ZYX** | LIM3_Zyxin, LIM superfamily, LIM2_Zyxin, PHA03247 superfamily | **PB0244** |
| **CAI9699750.1** | [NCBI](https://www.ncbi.nlm.nih.gov/protein/CAI9699750.1) | **CTNNB1** | CTNNAbd_CTNNB1, ARM, PHA03307 superfamily, SRP1 superfamily | **PB0245** |
| **CAI9708762.1** | [NCBI](https://www.ncbi.nlm.nih.gov/protein/CAI9708762.1) | **RAC1** | Rac1_like | **PB0246** |
| **CAI9710421.1** | [NCBI](https://www.ncbi.nlm.nih.gov/protein/CAI9710421.1) | **FERMT2** | PH_fermitin, FERM_F1_KIND2, FERM_F0_KIND2, PH-like superfamily, FERM_M, B41 superfamily | **PB0247** |

| **CAI9711810.1** | [NCBI](https://www.ncbi.nlm.nih.gov/protein/CAI9711810.1) | **VCL** | Vinculin | **PB0248** |
| --- | --- | --- | --- | --- |
| **CAI9698168.1** | [NCBI](https://www.ncbi.nlm.nih.gov/protein/CAI9698168.1) | **IBSP** | BSP_II | **PB0249** |
| **CAI9710417.1** | [NCBI](https://www.ncbi.nlm.nih.gov/protein/CAI9710417.1) | **BMP4** | TGF_beta_BMP4_BMP2B, TGFb_propeptide | **PB0251** |
